# Risk of using logistic regression to illustrate exposure-response relationship of infectious diseases

**DOI:** 10.1186/1471-2334-14-540

**Published:** 2014-10-04

**Authors:** Jinma Ren, Zhen Ning, Carmen S Kirkness, Carl V Asche, Huaping Wang

**Affiliations:** Center for Outcomes Research, University of Illinois College of Medicine at Peoria, One Illini Drive, Box 1649, Peoria, IL 61656 USA; Shanghai Municipal Center for Disease Control and Prevention, Shanghai, China; University of Illinois College of Pharmacy at Chicago, Peoria, IL USA

**Keywords:** Logistic regression, Measurement error, Infectious disease, Exposure-response relationship, Computer simulation

## Abstract

**Background:**

In most biological experiments, especially infectious disease, the exposure-response relationship is interrelated by a multitude of factors rather than many independent factors. Little is known about the suitability of ordinary, categorical exposures, and logarithmic transformation which have been presented in logistic regression models to assess the likelihood of an infectious disease as a function of a risk or exposure. This study aims to examine and compare the current approaches.

**Methods:**

A simulated human immunodeficiency virus (HIV) population, dynamic infection data for 100,000 individuals with 1% initial prevalence and 2% infectivity, was created. Using the Monte Carlo method (computational algorithm) to repeat random sampling to obtain numerical results, linearity between log odds and exposure, and suitability in practice were examined in the three model approaches.

**Results:**

Despite diverse population prevalence, the linearity was not satisfied between log odds and raw exposures. Logarithmic transformation of exposures improved the linearity to a certain extent, and categorical exposures satisfied the linear assumption (which was important for modelling). When the population prevalence was low (assumed < 10%), performances of the three models were significantly different. Comparing to ordinary logistic regression, the logarithmic transformation approach demonstrated better accuracy of estimation except that at the two inflection points: likelihood of infection increased from slowly to sharply, then slowly again. The approach using categorical exposures had better estimations around the real values, but the measurement was coarse due to categorization.

**Conclusions:**

It is not suitable to directly use ordinary logistic regression to explore the exposure-response relationship of HIV as an infectious disease. This study provides some recommendations for practical implementations including: 1) utilize categorical exposure if a large sample size and low population prevalence are provided; 2) utilize a logarithmic transformed exposure if the sample size is insufficient or the population prevalence is too high (such as 30%).

**Electronic supplementary material:**

The online version of this article (doi:10.1186/1471-2334-14-540) contains supplementary material, which is available to authorized users.

## Background

### Motivating example

Our motivating example arises from a study of the exposure-response relationship between the number of sex partners and prevalent HIV infection among men who have sex with men (MSM), leading to methodological challenges. Data were collected on 1,072 MSM from a retrospective epidemiological survey in Shanghai during the period between April 2008 and September 2009, including the binary response variable *Y* (current HIV status), a quantitative risk factor *X* (number of sex partners in the past 6 months), and other covariates *Z* (social-economic factors, pattern of sex partners and condom use).

**Figure 1:**
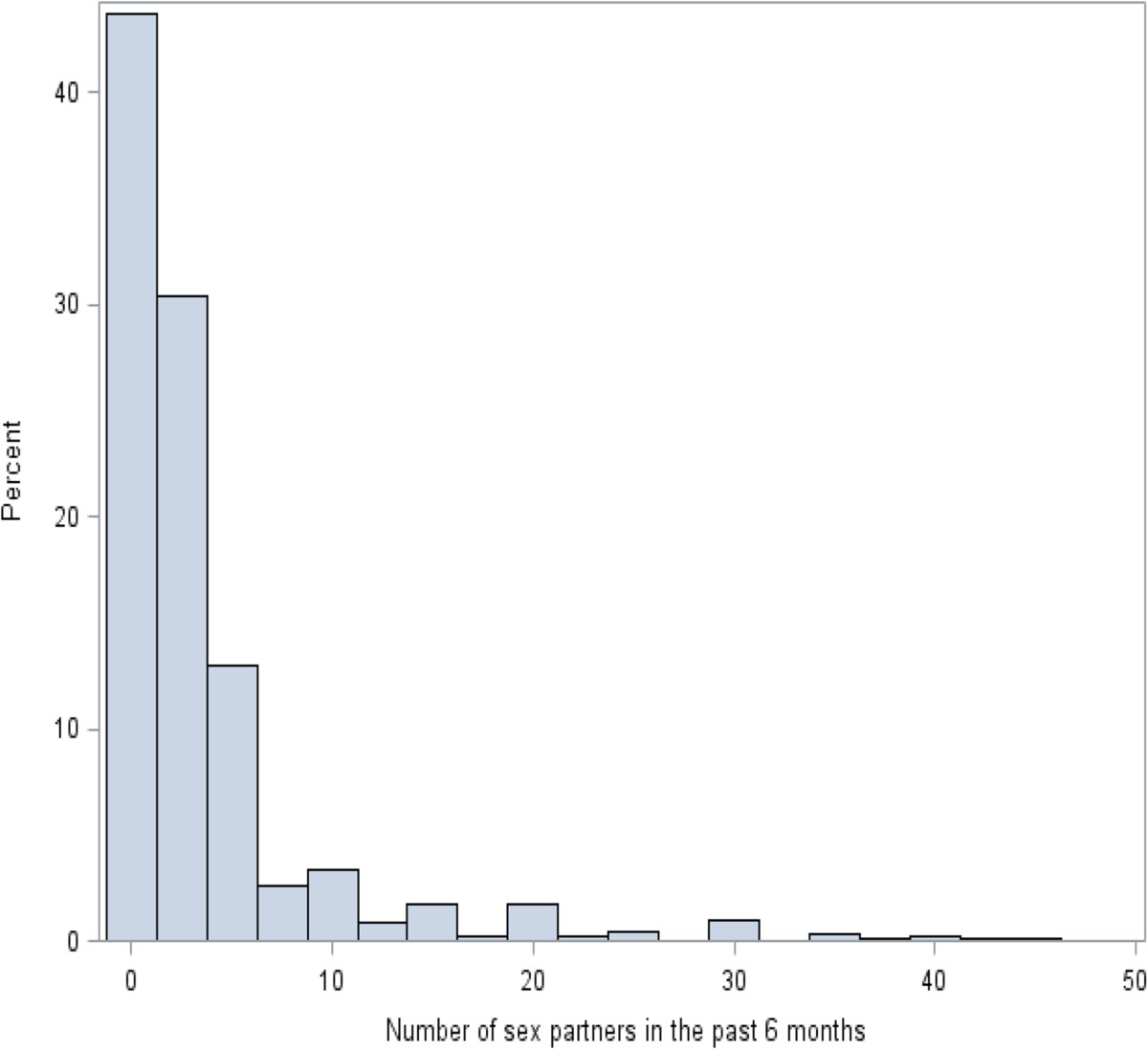
**Distribution of sex partners in the past six months (truncated by 50).**

The primary challenge in this analysis was the fact that the distribution of *X* was not normal, but approximately negative binomial (Figure 1). The negative binomial distribution supposes there is a sequence of independent Bernoulli trials (or binomial trials), each trial having two potential outcomes called “success” and “failure”. In each trial the probability of success is *p* and of failure is *(1-p)*. This sequence is observing until a predefined number *r* of failures has occurred. Then the observed random number of successes, *X*, will have the negative binomial distribution [1, 2]. In this study, the “success” and “failure” are the success and failure of having a sex partner at each contact, respectively. The probability function of negative binomial distribution is

$$f\left(k;r,p\right)\equiv \mathrm{Pr}\left(X=k\right)=\left(\begin{array}{c} k+r-1\\ k \end{array}\right)p^{k}{\left(1-p\right)}^{r}for\;k=0,1,2,\ldots$$

For this reason, it might fail to meet the linearity assumption between the log odds [*logitPr*(*Y* = 1|*X*)] and the continuous covariate (*X*) if logistic regression is used to analyze this data [3]. For this reason, two approaches were found in the previous studies: 1) group *X* into a categorical variable [4, 5], and 2) use of a logarithmic transformation of *ln(X + 1)*[6]. However, little is known about the accuracy and suitability of these approaches under these circumstances.

### Introduction

Logistic regression is widely used to assess the likelihood of an infectious disease as a function of a risk or exposure factor (and covariates), to illustrate the exposure-response relationship [4–8]. However, it should be noted that logistic regression assumes a linear relationship between independent variables and log odds [3]. Whilst it does not require the dependent and independent variables to be related linearly, it requires that the independent variables are linearly related to the log odds.

Do the studies of infectious disease satisfy the linear assumption? For infectious diseases, the exposure of individual is not independent yet, and the exposure-response relationship is an intricate net instead of independent factors. The data of exposure is usually present with a skewed distribution and heterogeneity of variance [9–13]. In our motivating example, it was an approximate negative binomial distribution. Thus, it is critical to examine the linear assumption before we apply logistic regression to analyze the exposure-response relation.

But, it is still unclear which approach has a better calibration, and minimizes the errors between the predicted values and the real data. Therefore, this study aims to compare the suitability of these approaches which have been broadly used to study the exposure-response relationship of infection disease using simulated and real-world data.

## Methods

### Simulation of infected population

We used PROC IML in SAS 9.3 to create a dynamic HIV infection model among 100,000 enclosed MSM individuals under certain conditions (Additional file 1). The initial prevalence and the infectivity rate in the population were set as 1% and 2%, respectively [4, 14]. The infectivity rate here could be a little higher than that in the real-world MSM population because we wanted to save the runtime of SAS. During an incubation period, the number of persons (including infected and non-infected) whom each individual could contact was assumed to be a negative binomial distribution (p = 0.1, r = 1) because we assumed the chance of exposure was not equal for everyone (such as HIV infection). The model was stopped when it reached a targeted prevalence (10%, 20%, 30% and 40%). The immunization, treatment and intervention were not considered in this simulation, so the disease status would be permanent once a person became infected. The simulation process is described as below:

We set a closed population with 100,000 individuals, and 1% of them with HIV positive.We randomly set a number of sex partners (exposure) for each individual based on a negative binomial distribution (p = 0.1, r = 1), so that the distribution of sex partners liked that Figure 1 showed.

A dynamic HIV infection started. Each person randomly selected his own sex partners according to the number of sex partners which was set in the step 2. For example, if a person was set 0 sex partner, he was not allowed to find any sex partner. If a person was set 2 sex partners, he could select two sex partners who were available. It called one generation when all individuals had reached the preset number of sex partners. Then a new generation started. A person was considered HIV positive when the individual had ≥50 sex contacts with HIV positive persons.

The model stopped when it reached the preset targeted prevalence (10%, 20%, 30% or 40%).

The outcome was a binary dependent *Y* (infection or non-infection), and the continuous covariate *X* was defined as the number of exposures (contact other persons) during an incubation period. We didn’t define *X* as the number of exact exposures (only contact patients) because we might not know whether the contacted persons are infected in the real world. Thus, based on this simulated data, the population exposure-response relationship between *Y* and *X* could be easily drawn. Also, it would be possible to examine the linear assumption between the log odds and the exposure variable.

### Logistic regression models with maximum likelihood estimation

We attempted three approaches of logistic regression to analyze the simulated data as below.

Model A: Raw *X* was used as a predictor to estimate the parameter (*β*), i.e.,

$$\mathrm{logitPr}\left(Y=1|X\right)=\alpha +\beta X$$

Model B: A categorical variable *X*_*c*_ was put in the model instead of raw *X*, which was grouped as 0-2, 3-5, 6-10, 11-15, 16-20, 21-25, 26-30, 31-35, 36-40 and > =41. The function was similar with the model A,

$$\mathrm{logitPr}\left(Y=1|X\right)=\alpha +\beta X_{c}$$

where *X*_*c*_ was considered as a dummy/nominal variable.

Model C: A logarithmic transformation was used to transform raw *X*, i.e.,

$$\mathrm{logitPr}\left(Y=1|X\right)=\alpha +\beta \ln\left(X+1\right)$$

### Monte Carlo experiment for comparison of models

We repeated the simulation 3000 times to randomly select 10% of population. In order to estimate the population parameter (*β*), the three models (A, B and C) were all run for each sample, then each model had 3000 sample statistics (*β*). PROC LOGISTIC, PROC SQL and %MACRO in SAS 9.3 were used for this experiment (Additional file 2).

The means and standard deviations of sample statistics *β*_*s*_ were recorded, and the predicted probability (likelihood of HIV infection given a certain number of sex partners) was scored by each model. A model would be considered better if it could satisfy the following criteria: 1) predicted values were closer to the true values, and 2) smaller standard deviation.

### Real-world data for validation

This study also used real-world data to validate the findings of simulation. A total of 1,072 MSM were recruited in Shanghai through the snowball sampling method [15] during the period between April 2008 and September 2009.

The survey questionnaire was based on that used in the National Sentinel Surveillance Program since 1995, with modifications based on Chinese MSM community feedback. Local Centers for Disease Control and Prevention (CDC) staff who conducted the surveys by interview were given intensive training and a detailed protocol. Interview settings had at least one private interview/counseling room, a testing room, and a waiting room, and were usually located within a hospital or local CDC facility. Blood samples were collected from each subject, and tested in the laboratory of Shanghai CDC for HIV. Counseling was provided before and after testing. Participants who tested negative were noticed by local CDC staff, whereas those who tested positive were referred to the National AIDS Program and/or a local hospital or clinic.

The primary measure of this study was to examine the prevalence changes of HIV (outcome) along with an increase of the number of sex partners (exposure), controlling other confounders (such as age, marital status, race, highest education level achieved, monthly income, self-identified sexual orientation, condom use, commercial sex behavior and sexual activity with a female).

### Ethical approval

The real-world data in this study was the Shanghai component of the national cross-sectional survey of 61 cities in China [4]. The national survey was reviewed and approved by the Institutional Review Board of the National Center for AIDS/STD Control and Prevention, China CDC. All participants provided informed consent that information from surveys and blood tests could be used for scientific studies when they were recruited, and all the data in the study were de-identified. So, this study is in compliance with the Helsinki Declaration.

## Results

### Target exposure-response curves

**Figure 2:**
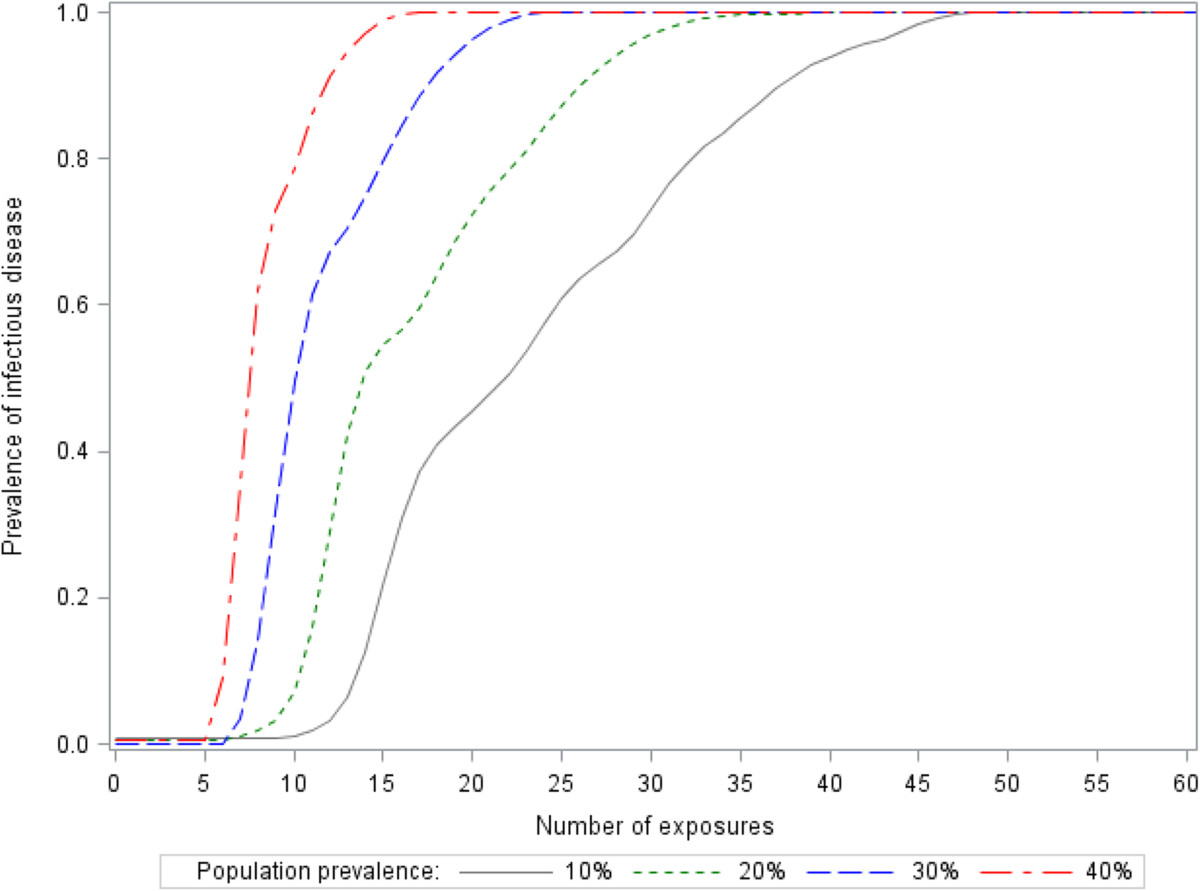
**Simulated infections among 100,000 enclosed individuals.** Initial prevalence 1% and infectivity rate 2% were set. During an incubation period, the number of persons (including infected and non-infected) whom each individual could contact was assumed to be negative binomial (p = 0.1, r = 1). The model was paused when it reached a targeted prevalence (10%, 20%, 30% and 40%).

As Figure 2 depicted, the prevalence of infection immediately jumped up when the exposure was up to a certain number. Given population prevalence 30%, the risk of infection increased rapidly at seven exposures, and approached the asymptote 100% at twenty exposures. Overall, the exposure-response relationship was an approximate generalized logistic curve.

### Linear assumption of logistic regression

**Figure 3:**
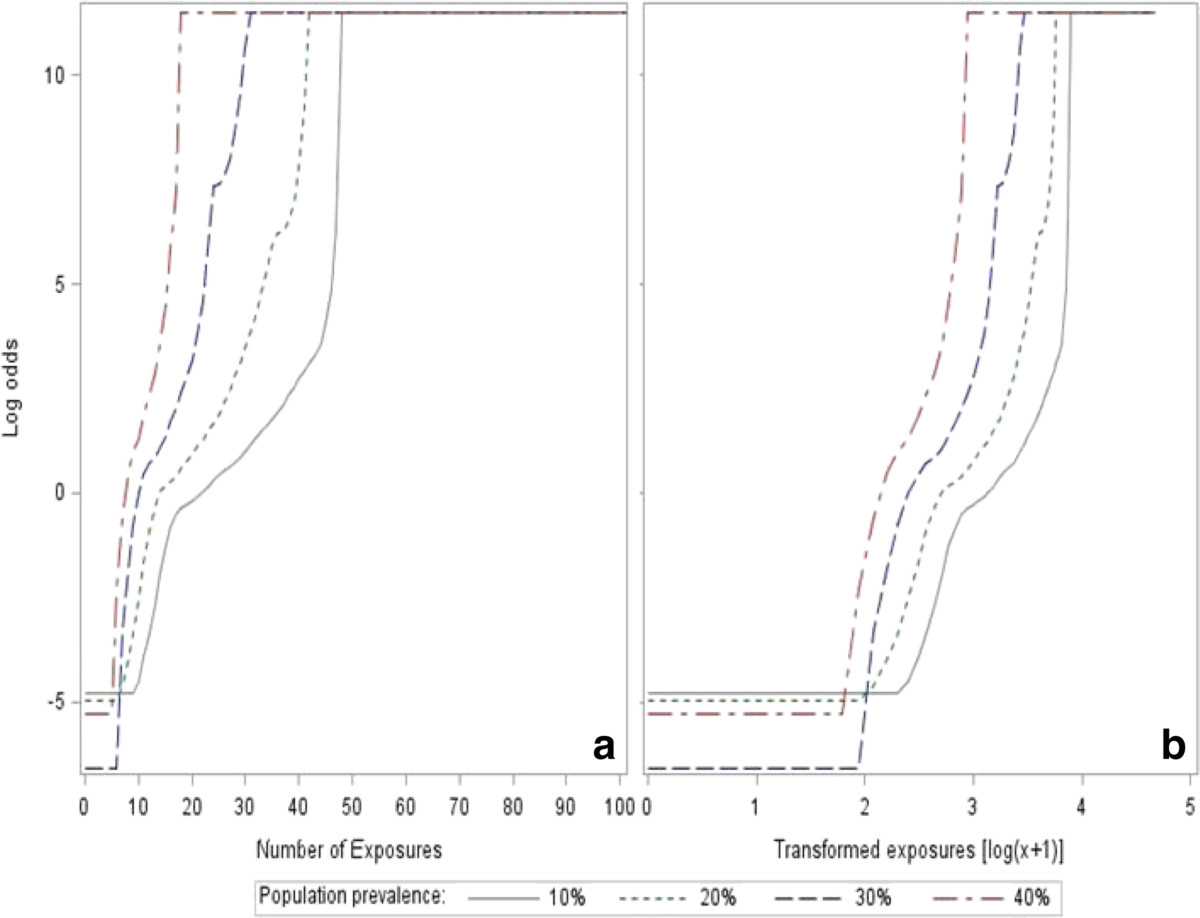
**Linear assumption of logistic regression. (a)** indicated the relationship of log odds and exposures, whereas **(b)** showed the relationship of log odds and transformed exposures [log(x + 1)] instead of real exposures.

Despite diverse population prevalence, the linearity was not satisfied between the log odds and exposures (Figure 3a). Although it improved the linearity in a certain extent, the logarithmic transformation of exposures still failed to deal with the linear issue when the log odds were extremely small or large (Figure 3b).

To categorize, the exposure was another way of addressing linearity. Linear assumption of logistic regression was satisfied when the exposure factor was measured as a categorical/dummy variable.

### Comparison of logistic regression models

**Figure 4:**
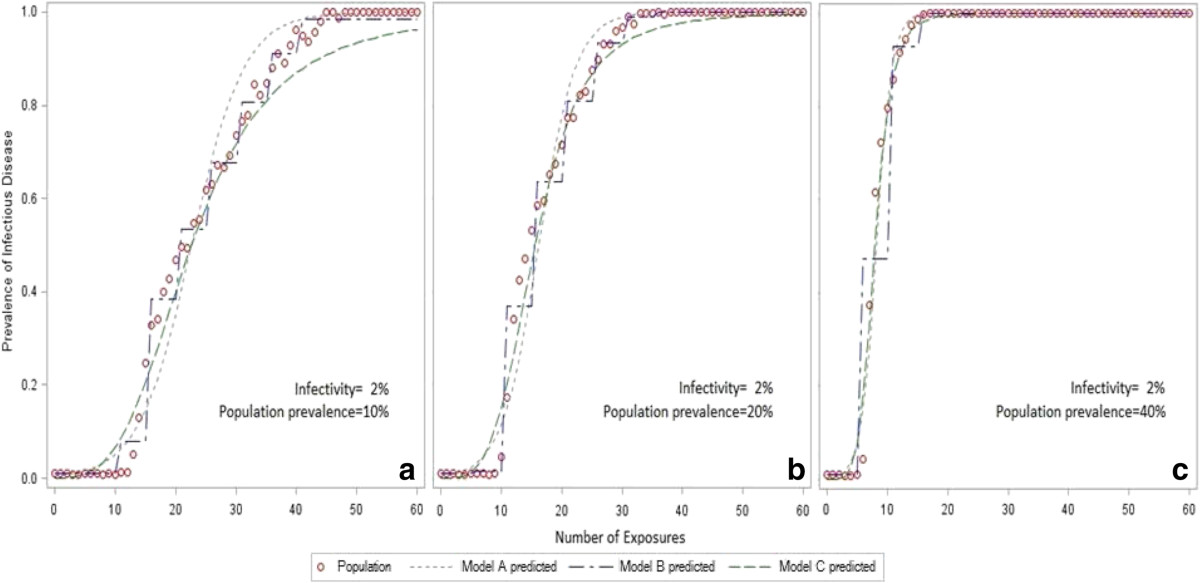
**Comparison of logistic regression models based on simulated data.** The predictor (number of exposures) was raw, categorical and transformed exposures in the model A, B and C, respectively. **(a)** indicated that the infectivity was 2% and the population prevalence was 10%, **(b)** indicated that the infectivity was 2% and the population prevalence was 20%, whereas **(c)** indicated that the infectivity was 2% and the population prevalence was 40%.

As Figure 4 depicted, the performances of the three models were very close when the population prevalence was up to 40%. However, they were significantly different when the population prevalence was low, such as 10%.All the three models worked very well when the exposure-response relationship was at the rising phase, where the risk of infection was increased sharply (Figure 4a,b). Given a low population prevalence, Model B demonstrated its advantages at the inflection points of the curves comparing to Model A and Model C. However, the measurement of Model B was coarse due to categorization. Overall, Model C showed reasonable accuracy of estimation except at the two inflection points.

Table 1 also indicated that Model B had higher variability because the coefficients of variation were greater compared to Model A and Model C. Direct comparisons of the model coefficient was not possible due to the difference in units and transformations.

**Table 1:** **Means and standard deviations of 3000 sample statistics (HIV prevalence = 10%)**

Models	Parameters	Mean	Standard deviation	Coefficient of variation
**A (Ordinary)**	Intercept (*α*)	−5.24	0.10	−2%
	Coefficient (*β*)	0.23	0.01	4%
**B (Categorical)**	Intercept (*α*)	−0.66	0.41	−62%
0-2 partners	Coefficient (*β*)	Ref	Ref	
3-5 partners	Coefficient (*β*)	−0.14	0.29	−207%
6-10 partners	Coefficient (*β*)	−0.14	0.28	−200%
11-15 partners	Coefficient (*β*)	2.06	0.52	25%
16-20 partners	Coefficient (*β*)	4.06	0.52	13%
21-25 partners	Coefficient (*β*)	4.67	0.53	11%
26-30 partners	Coefficient (*β*)	5.26	0.53	10%
31-35 partners	Coefficient (*β*)	5.97	0.55	9%
36-40 partners	Coefficient (*β*)	6.91	0.73	11%
> = 41 partners	Coefficient (*β*)	10.02	3.78	38%
**C (Log transformation)**	Intercept (*α*)	10.95	0.46	−4%
	Coefficient (*β*)	3.46	0.16	5%

### Validation of real-world data

**Figure 5:**
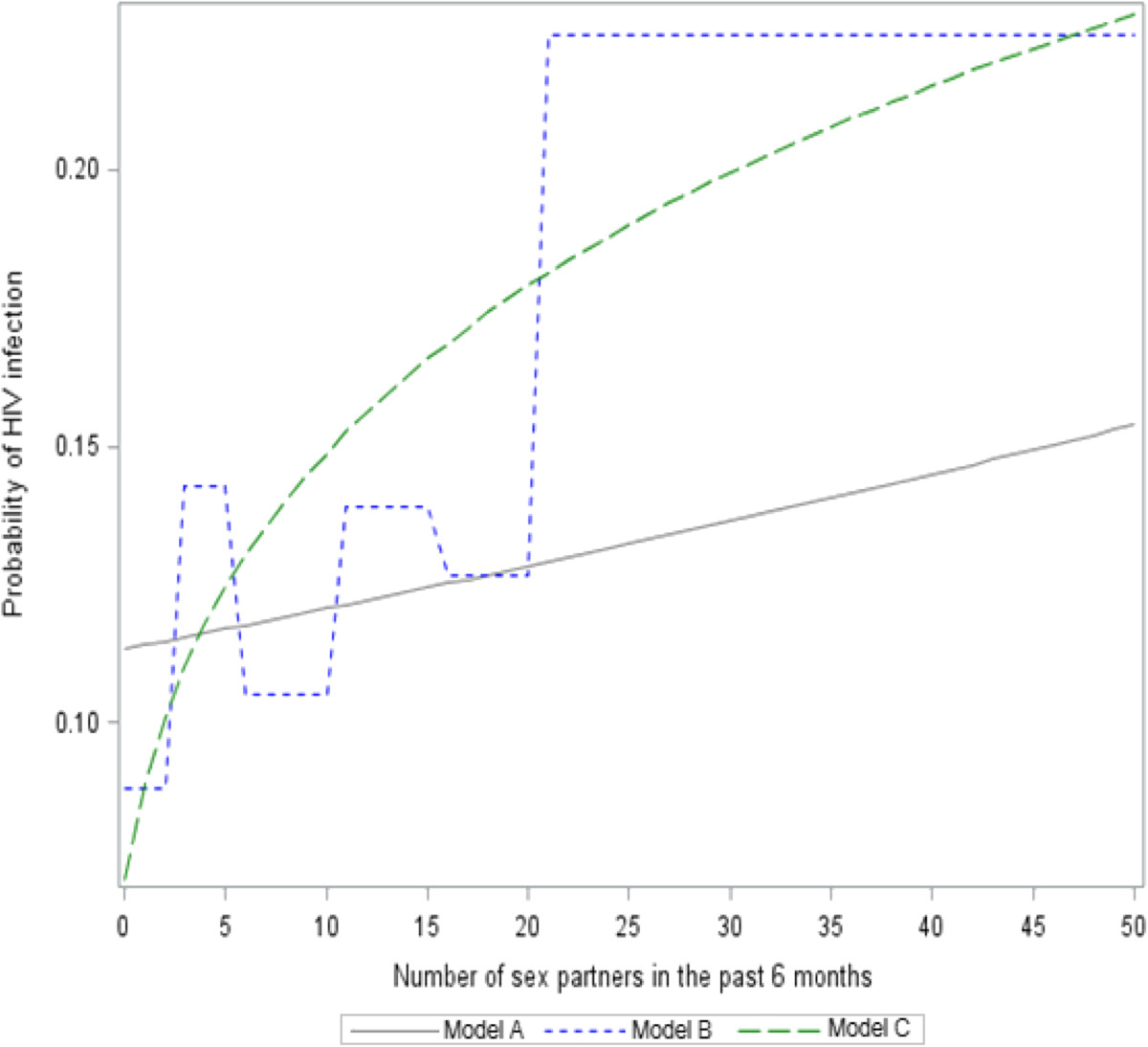
**Comparison of logistic regression models based on real-world data about HIV infection.** The predictor (number of exposures) was raw, categorical and transformed exposures in the model A, B and C, respectively.

The prevalence of HIV was 7.8% among this MSM population. As Figure 5 depicted, Model B and Model C had a similar trend of prediction in the real-world data, but Model B fluctuated greatly. The relationship between HIV infection and number of sex partners was almost linear in Model A, which was far away from the truth. Overall, it supported our findings in the simulated data.

Meanwhile, we found that the exposure-response curves in the real-world data were different from that in the simulated data. The former was an approximate exponential curve, and the latter was a generalized logistic curve.

## Discussion and conclusion

This study focused on assessing the risk of using logistic regression to illustrate an exposure-response relationship of HIV as an infectious disease, which is different from previous simulation studies discussed diverse measurement errors in logistic regression [16–21]. Logistic regression requires a linearity between independent variables and log odds [3]. However, this study found that the linear assumption usually could not be satisfied when an ordinary logistic regression was used to explore the exposure-response relationship of HIV as an infectious disease. Although it could improve the linearity in a certain extent, logarithmic transformation might not correct the linearity when the exposure is very little or huge. So, the performance of these logistic regression models would certainly be affected by the non-linear circumstance. In order to overcome the linear issue, categorical exposures (dummy variables) are used in logistic regression because dummy variables are only expressed by 0 and 1.In this study, we found that the non-linear circumstance (two inflection points) mainly affected the prediction of Model A (raw exposure) and Model C (logarithmic transformation) when the population prevalence was low. If the prevalence rate is high, individuals could be more likely to be infected even if they have a few exposures, so that the infectious disease could spread quickly. That is to say, the first inflection point in Figure 3 should be closer to zero, which would improve the linearity. Therefore, it is not surprise to see the three models are very similar in Figure 4c.

It was proved that Model B (categorical exposure) was not related to the linear assumption in this study. So, Model B could get appropriate estimates whatever the population rate would be. However, Model B is not a perfect solution either, because different studies could have different categorical rules and we might have to use a coarse category due to a small sample size. And it should be noted that an inappropriate categorization could significantly underestimate or overestimate the real odds ratio.

In this study, the real-world data about HIV infection among MSM supported our findings in the simulated data overall, but we also found that the exposure-response curves were obviously different between the two data. The reason could be related to more risk factors and confounders in the real-world data. We could not get the same benchmark between the two data, although the logistic regression models adjusted some confounders (such as demographics and sexual behaviors). The simulated data was pure because the number of exposures was a risk factor only. But, there are many known and unknown factors which could affect HIV infection in the real world even if there were no sex partners in the past six months.

This study provides lots of valuable findings, nevertheless there are limitations to consider when the results are interpreted. Primarily, the simulated data couldn’t consider all circumstances (such as observation errors), so this study only simulated different population prevalence with fixed infectivity and other conditions. Secondly, this study still couldn’t provide an optimal solution about this linear issue, but some recommendations for practical implementations could be concluded: 1) utilize categorical exposure if a large sample size and low population prevalence are provided; 2) utilize a logarithmic transformed exposure if the sample size is insufficient or the population prevalence is too high (such as 30%).

## Electronic supplementary material

Additional file 1: SAS codes for simulation of infected population.(DOCX 29 KB)

Additional file 2: SAS codes for comparison of logistic regress models.(DOCX 27 KB)

Below are the links to the authors’ original submitted files for images.

Authors’ original file for figure 1

Authors’ original file for figure 2

Authors’ original file for figure 3

Authors’ original file for figure 4

Authors’ original file for figure 5

Authors’ original file for figure 6
