# Supplementary material for: Risk of using logistic regression to illustrate exposure-response relationship of infectious diseases
Source: BMC Infect Dis. 2014 Oct 4;14:540. doi: 10.1186/1471-2334-14-540 (PMC4287313; doi:10.1186/1471-2334-14-540)
Supplement: Supplementary file 1 — Additional file 1: SAS codes for simulation of infected population.(DOCX 29 KB) [file 12879_2014_3848_MOESM1_ESM.docx]

Additional file 1: SAS codes for simulation of infected population

/****************************************************************************

*Initial prevalence: P0=1%

*Infectivity: 2% (onset after 50 exposures);

*Contact probability distribution: negative binomial (p=0.1, r=1)

*Targeted prevalence: P1=10%, 20%, 30% and 40%;

****************************************************************************/

libname rjm "U:\Jinma Ren\WORK\UIC\JINMA\STI simulation\Data";

**%macro** simulation(population, P0, P1, Infectivity);

proc iml;

*0) Define the population;

pop=do(**1**,&population,**1**)`;

n=countn(pop,"COL");

d0=J(n[**1**,**1**],**2**,**0**);

c=J(n[**1**,**1**],**1**,**0**);

c_all=J(n[**1**,**1**],**1**,**0**);

gen=J(n[**1**,**1**],**1**,**0**);

*1) Create the first generation cases;

do k=**1** to n[**1**,**1**];

*Initial prevalence 1%;

if ranuni(**2013**)<&P0 then d0[k,**1**]=**1**;

call RANDSEED(**2013**);

*Contact probability distribution is negative binomial (p=0.1, r=1);

call RANDGEN(c,'negbinomial',**0.1**,**1**);

end;

*2) Start infection;

do g=**1** to **1000**; *Maximum of generations;

c1=J(n[**1**,**1**],**1**,**0**); *Count the effective contacts in each generation;

do i=**1** to n[**1**,**1**];

if c[i,**1**]=**0** then d0[i,**1**]=d0[i,**1**];

if c[i,**1**]^=**0** & c[i,**1**]>c1[i,**1**] then do;

do j=**1** to **10000**;

*Randomly selecting individuals (only go forward);

r=J(**1**,**1**,int(ranuni(**2013**+j)*(n[**1**,**1**]-i))+i+**1**);

*Same situation, case-case or control-control;

if r^=i & d0[i,**1**]=d0[r,**1**] & c[r,**1**]>c1[r,**1**] then do;

c1[i,**1**]=c1[i,**1**]+**1**;

c1[r,**1**]=c1[r,**1**]+**1**;

if d0[i,**1**]=**1** then d0[i,**2**]=d0[i,**2**]+**1**;

if d0[r,**1**]=**1** then d0[r,**2**]=d0[r,**2**]+**1**;

c_all[i,**1**]=c_all[i,**1**]+**1**;

c_all[r,**1**]=c_all[r,**1**]+**1**;

end;

*Different situation, case-control;

if r^=i & d0[i,**1**]=**1** & d0[r,**1**]=**0** & c[r,**1**]>c1[r,**1**] then do;

c1[i,**1**]=c1[i,**1**]+**1**;

c1[r,**1**]=c1[r,**1**]+**1**;

d0[r,**2**]=d0[r,**2**]+**1**;

c_all[i,**1**]=c_all[i,**1**]+**1**;

c_all[r,**1**]=c_all[r,**1**]+**1**;

end;

*Different situation, control-case;

if r^=i & d0[i,**1**]=**0** & d0[r,**1**]=**1** & c[r,**1**]>c1[r,**1**] then do;

c1[i,**1**]=c1[i,**1**]+**1**;

c1[r,**1**]=c1[r,**1**]+**1**;

d0[i,**2**]=d0[i,**2**]+**1**;

c_all[i,**1**]=c_all[i,**1**]+**1**;

c_all[r,**1**]=c_all[r,**1**]+**1**;

end;

*Infectivity: get sick after n exposures;

if d0[i,**2**]>=&Infectivity then d0[i,**1**]=**1**;

if d0[r,**2**]>=&Infectivity then d0[r,**1**]=**1**;

if c[i,**1**]=c1[i,**1**] then goto Skip;

end;

Skip:

end;

end;

gen[,**1**]=gen[,**1**]+**1**;

if (d0[+,**1**]/&population)>=&P1 then stop;

end;

*3) Output the dataset;

out=d0||c||c1||c_all||gen;

create out var{case, exposure, contact_0, contact_1, contact_total, generation};

append from out;

quit;

**%mend** simulation;

%***simulation***(**100000**, **0.01**, **0.10**, **50**);

**data** rjm.data_10_50; set out; **run;**

%***simulation***(**100000**, **0.01**, **0.20**, **50**);

**data** rjm.data_20_50; set out; **run;**

%***simulation***(**100000**, **0.01**, **0.30**, **50**);

**data** rjm.data_30_50; set out; **run;**

%***simulation***(**100000**, **0.01**, **0.40**, **50**);

**data** rjm.data_40_50; set out; **run;**

/****************************************************************************

*Nonlinear fitness & Graph using the simulated data

****************************************************************************/

libname rjm "U:\Jinma Ren\WORK\UIC\JINMA\STI simulation\Data";

**%macro** score(data, data_out, knot);

ods output CrossTabFreqs=Crosstab;

proc freq data=&data;

table contact_1*case/nopercent norow nocol;

run;

data crosstab;

set crosstab;

sick=case;

run;

proc sql;

create table score as

select a.contact_1 as n, a.sick, a.frequency as y1, b.frequency as y0, (a.frequency/b.frequency) as y format=**9.4** from

(select contact_1, sick, frequency from Crosstab

where sick=**1** and contact_1~=**.**) a

left join

(select contact_1, sick, frequency from Crosstab

where sick=**.** and contact_1~=**.**) b

on a.contact_1=b.contact_1

order by a.contact_1;

quit;

ods graphics on;

proc transreg data=score;

model identity(y)=mspline(n/nknots=&knot);

output out=&data_out DAPPROXIMATIONS;

run;

proc sgplot data=&data_out;

scatter y=y x=n;

series y=Ay x=n;

run;

**%mend** score;

%***score***(rjm.data_10_50, rjm.data_10_50_out, **30**);

%***score***(rjm.data_20_50, rjm.data_20_50_out, **30**);

%***score***(rjm.data_30_50, rjm.data_30_50_out, **30**);

%***score***(rjm.data_40_50, rjm.data_40_50_out, **50**);
