# Supplementary material for: Risk of using logistic regression to illustrate exposure-response relationship of infectious diseases
Source: BMC Infect Dis. 2014 Oct 4;14:540. doi: 10.1186/1471-2334-14-540 (PMC4287313; doi:10.1186/1471-2334-14-540)
Supplement: Supplementary file 2 — Additional file 2: SAS codes for comparison of logistic regress models.(DOCX 27 KB) [file 12879_2014_3848_MOESM2_ESM.docx]

Additional file 2: SAS codes for comparison of logistic regress models

libname rjm "U:\Jinma Ren\WORK\UIC\JINMA\STI simulation\Data";

options nosource nonotes errors=**0**; *Suppress the log output file;

**%macro** sample(num, data, data_score);

proc sql;

create table OUT_all (y num format=**9.4**, ay num format=**9.4**, n num format=**9.0**, P_1 num format=**9.4**,

LCL_1 num format=**9.4**, UCL_1 num format=**9.4**, model num format=**9.0**, sample num format=**9.0**);

run;

proc sql;

create table PARA_all (variable char(**20**), DF num format=**9.0**, Estimate num format=**9.4**, StdErr num format=**9.4**, WaldChiSq num format=**9.4**, ProbChiSq num format=**9.4**, _ESTTYPE_ char(**20**), model num format=**9.0**, sample num format=**9.0**, Category num format=**9.0** );

run;

%do i=**1** %to &num;

data s&i;

set &data;

ran=ranuni(&i);

if ran<**0.90** then delete;

if contact_1<=**2** then contact_g=**1**;

if **3**<=contact_1<=**5** then contact_g=**2**;

if **6**<=contact_1<=**10** then contact_g=**3**;

if **11**<=contact_1<=**15** then contact_g=**4**;

if **16**<=contact_1<=**20** then contact_g=**5**;

if **21**<=contact_1<=**25** then contact_g=**6**;

if **26**<=contact_1<=**30** then contact_g=**7**;

if **31**<=contact_1<=**35** then contact_g=**8**;

if **36**<=contact_1<=**40** then contact_g=**9**;

if **41**<=contact_1 then contact_g=**10**;

log_contact_1=log(contact_1+**1**); *Tranformation1 of exposure;

run;

data &data_score;

set &data_score;

contact_1=n;

if contact_1<=**2** then contact_g=**1**;

if **3**<=contact_1<=**5** then contact_g=**2**;

if **6**<=contact_1<=**10** then contact_g=**3**;

if **11**<=contact_1<=**15** then contact_g=**4**;

if **16**<=contact_1<=**20** then contact_g=**5**;

if **21**<=contact_1<=**25** then contact_g=**6**;

if **26**<=contact_1<=**30** then contact_g=**7**;

if **31**<=contact_1<=**35** then contact_g=**8**;

if **36**<=contact_1<=**40** then contact_g=**9**;

if **41**<=contact_1 then contact_g=**10**;

log_contact_1=log(contact_1+**1**); *Tranformation1 of exposure;

run;

proc logistic data=s&i descend;

model case=contact_1;

ods output parameterestimates=para;

score data=&data_score out=out CLM;

run;

data para; set para; model=**1**; sample=&i; category=**.**; run;

data out; set out; model=**1**; sample=&i; keep ay y n P_1 LCL_1 UCL_1 model sample; run;

proc logistic data=s&i descend;

model case=log_contact_1;

ods output parameterestimates=para_log;

score data=&data_score out=out_log CLM;

run;

data para_log; set para_log; model=**2**; sample=&i; category=**.**; run;

data out_log; set out_log; model=**2**; sample=&i; keep ay y n P_1 LCL_1 UCL_1 model sample; run;

proc logistic data=s&i descend;

class contact_g(ref="1")/param=ref;

model case=contact_g;

ods output parameterestimates=para_cat;

score data=&data_score out=out_cat CLM;

run;

data para_cat; set para_cat; model=**3**; sample=&i; category=input(ClassVal0, **9.0**); drop ClassVal0; run;

data out_cat; set out_cat; model=**3**; sample=&i; keep ay y n P_1 LCL_1 UCL_1 model sample; run;

proc sql;

insert into PARA_all select * from para;

insert into PARA_all select * from para_log;

insert into PARA_all select * from para_cat;

run;

proc sql;

insert into OUT_all select * from out;

insert into OUT_all select * from out_log;

insert into OUT_all select * from out_cat;

run;

%end;

**%mend** sample;

%***sample***(**3000**, rjm.data_10_50, rjm.data_10_50_out);

**data** rjm.data_10_50_para_all; set para_all; **run**;

**data** rjm.data_10_50_out_all; set out_all; diff_abs=abs(ay-P_1); diff_pct=abs(ay-p_1)/ay***100**;**run**;

%***sample***(**3000**, rjm.data_20_50, rjm.data_20_50_out);

**data** rjm.data_20_50_para_all; set para_all; **run**;

**data** rjm.data_20_50_out_all; set out_all; diff_abs=abs(ay-P_1); diff_pct=abs(ay-p_1)/ay***100**;**run**;

%***sample***(**3000**, rjm.data_30_50, rjm.data_30_50_out);

**data** rjm.data_30_50_para_all; set para_all; **run**;

**data** rjm.data_30_50_out_all; set out_all; diff_abs=abs(ay-P_1); diff_pct=abs(ay-p_1)/ay***100**;**run**;

%***sample***(**3000**, rjm.data_40_50, rjm.data_40_50_out);

**data** rjm.data_40_50_para_all; set para_all; **run**;

**data** rjm.data_40_50_out_all; set out_all; diff_abs=abs(ay-P_1); diff_pct=abs(ay-p_1)/ay***100**;**run**;
