# Supplementary figures and images for: Risk of using logistic regression to illustrate exposure-response relationship of infectious diseases
Source: BMC Infect Dis. 2014 Oct 4;14:540. doi: 10.1186/1471-2334-14-540 (PMC4287313; doi:10.1186/1471-2334-14-540)

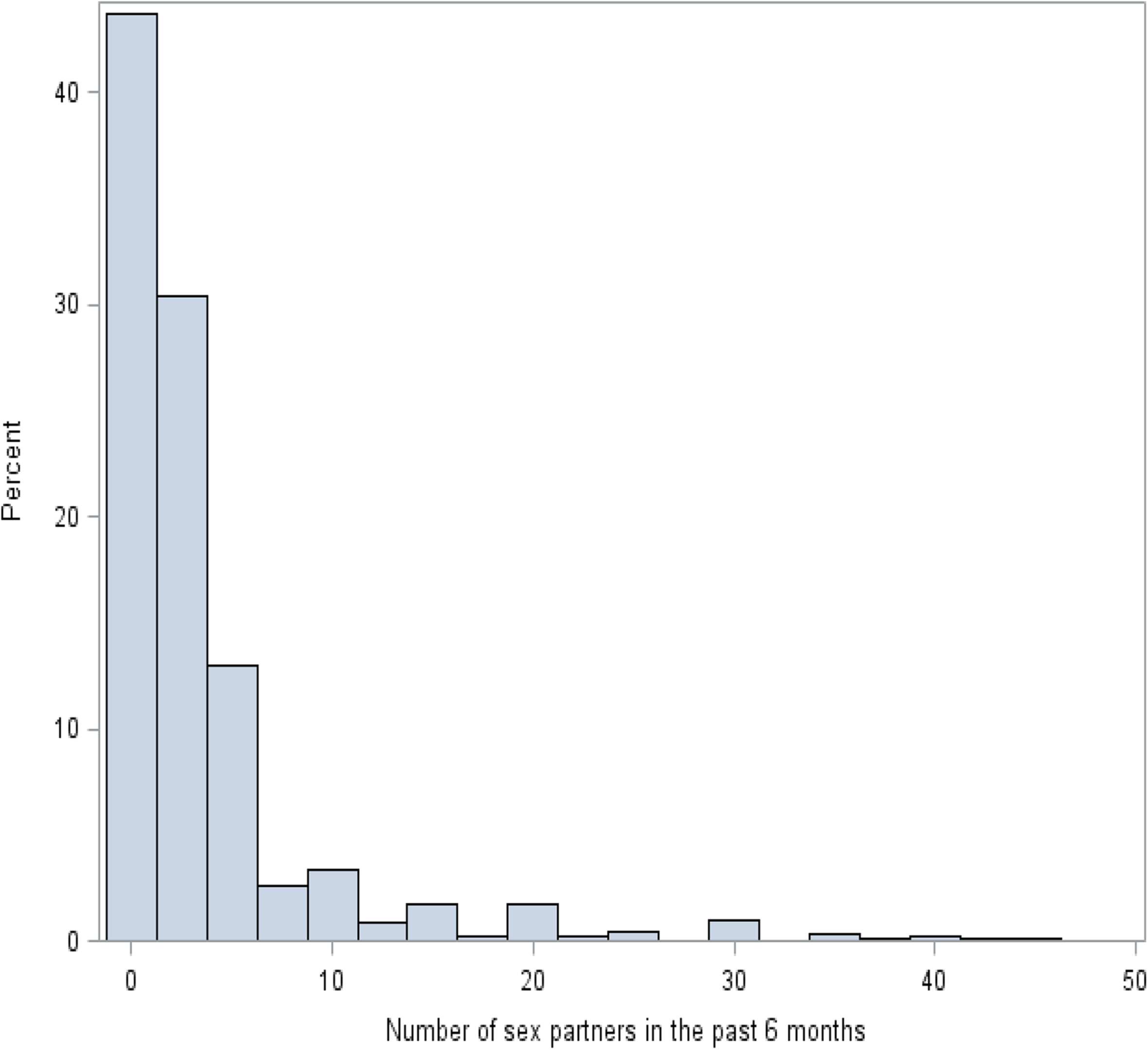

Supplement: Supplementary file 3 — Authors’ original file for figure 1 [file 12879_2014_3848_MOESM3_ESM.tif]

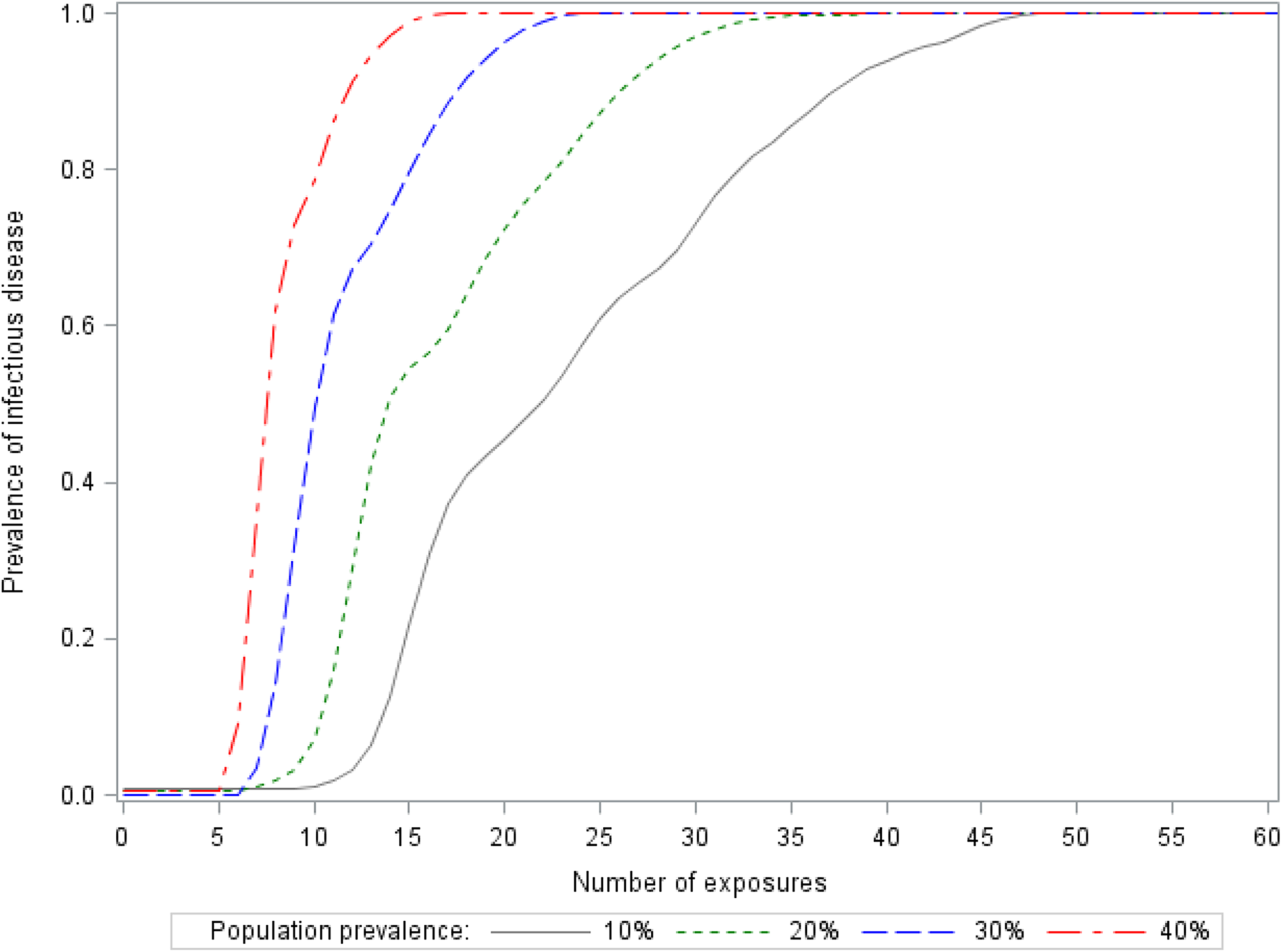

Supplement: Supplementary file 4 — Authors’ original file for figure 2 [file 12879_2014_3848_MOESM4_ESM.tif]

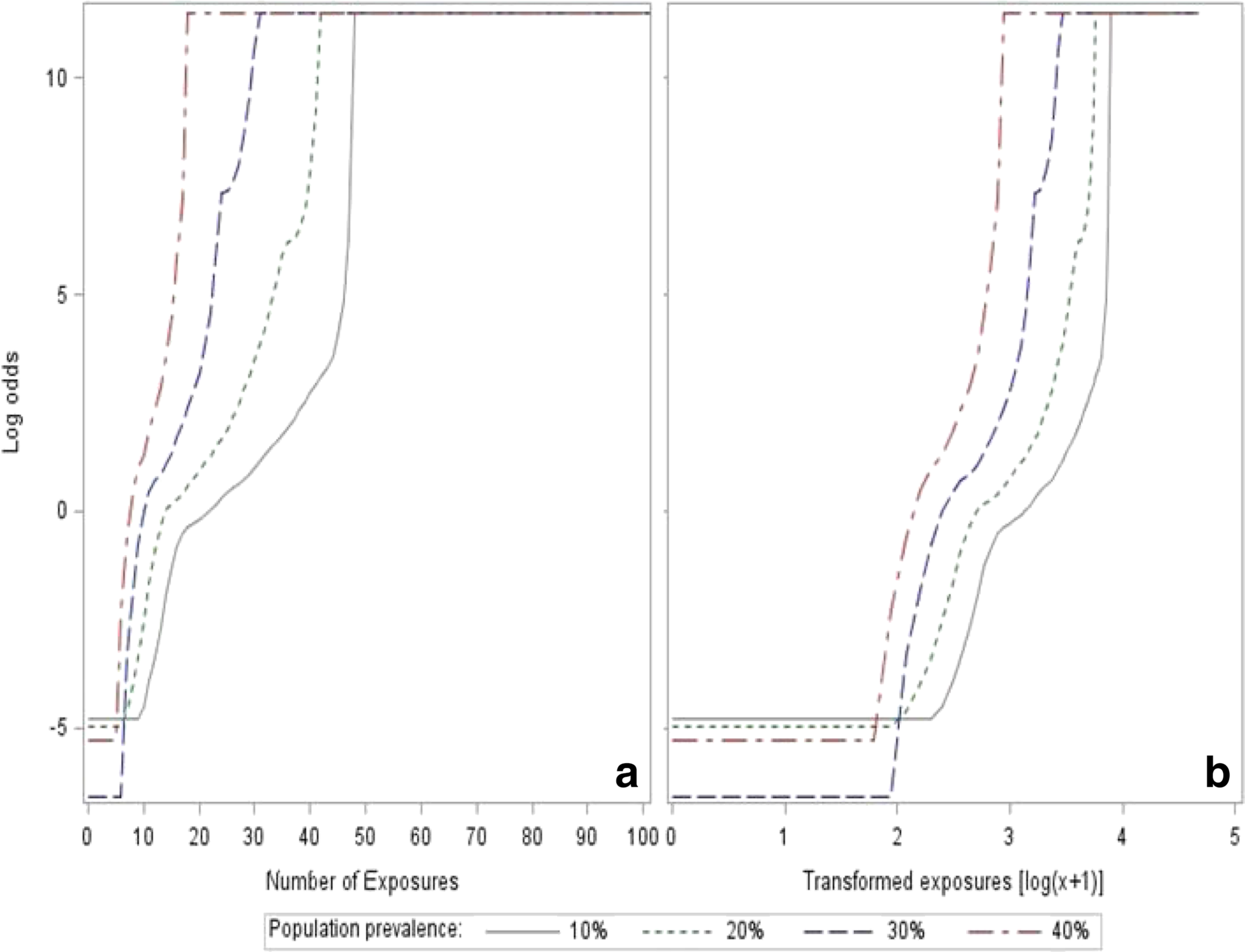

Supplement: Supplementary file 5 — Authors’ original file for figure 3 [file 12879_2014_3848_MOESM5_ESM.tif]

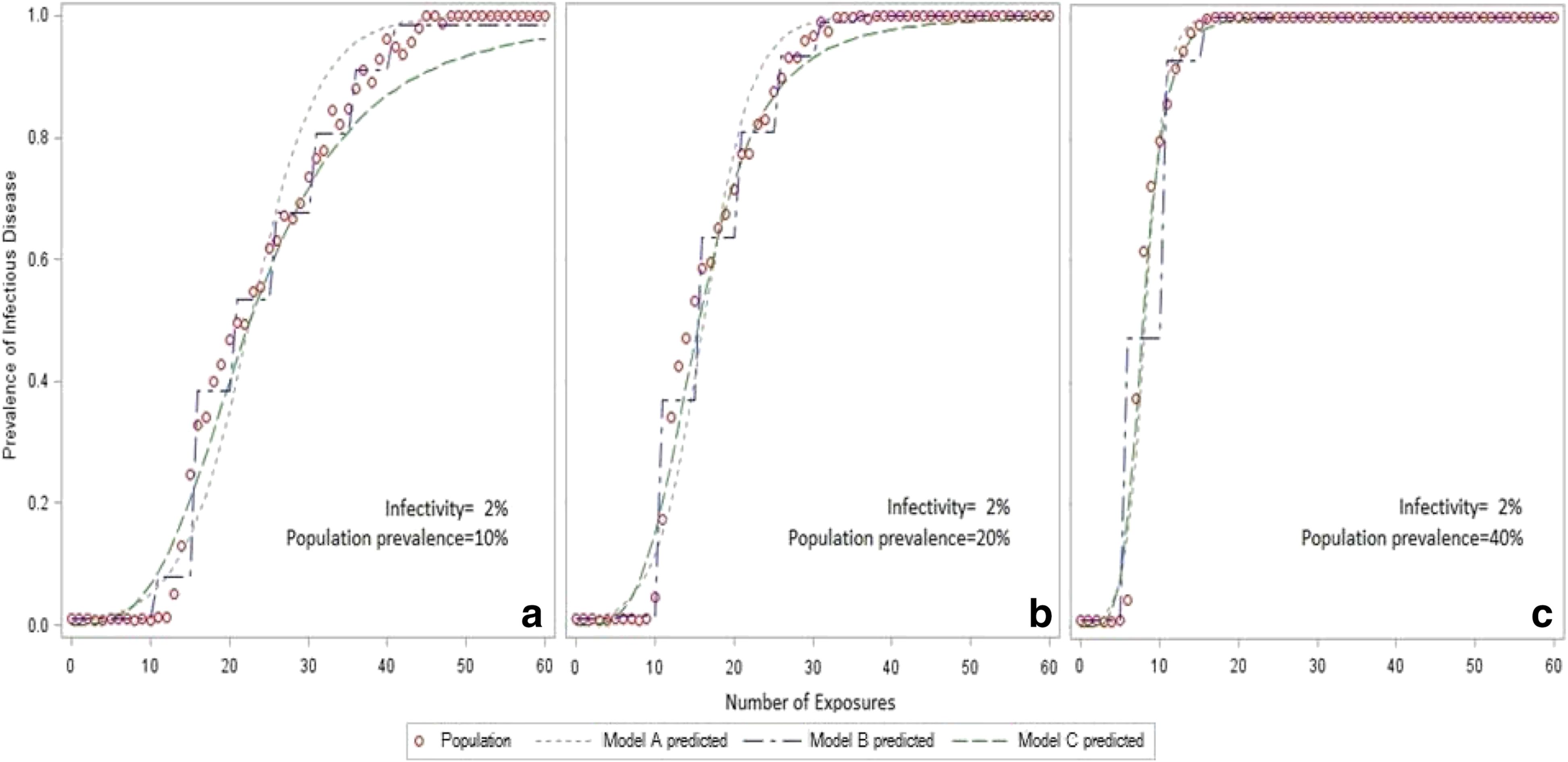

Supplement: Supplementary file 6 — Authors’ original file for figure 4 [file 12879_2014_3848_MOESM6_ESM.tif]

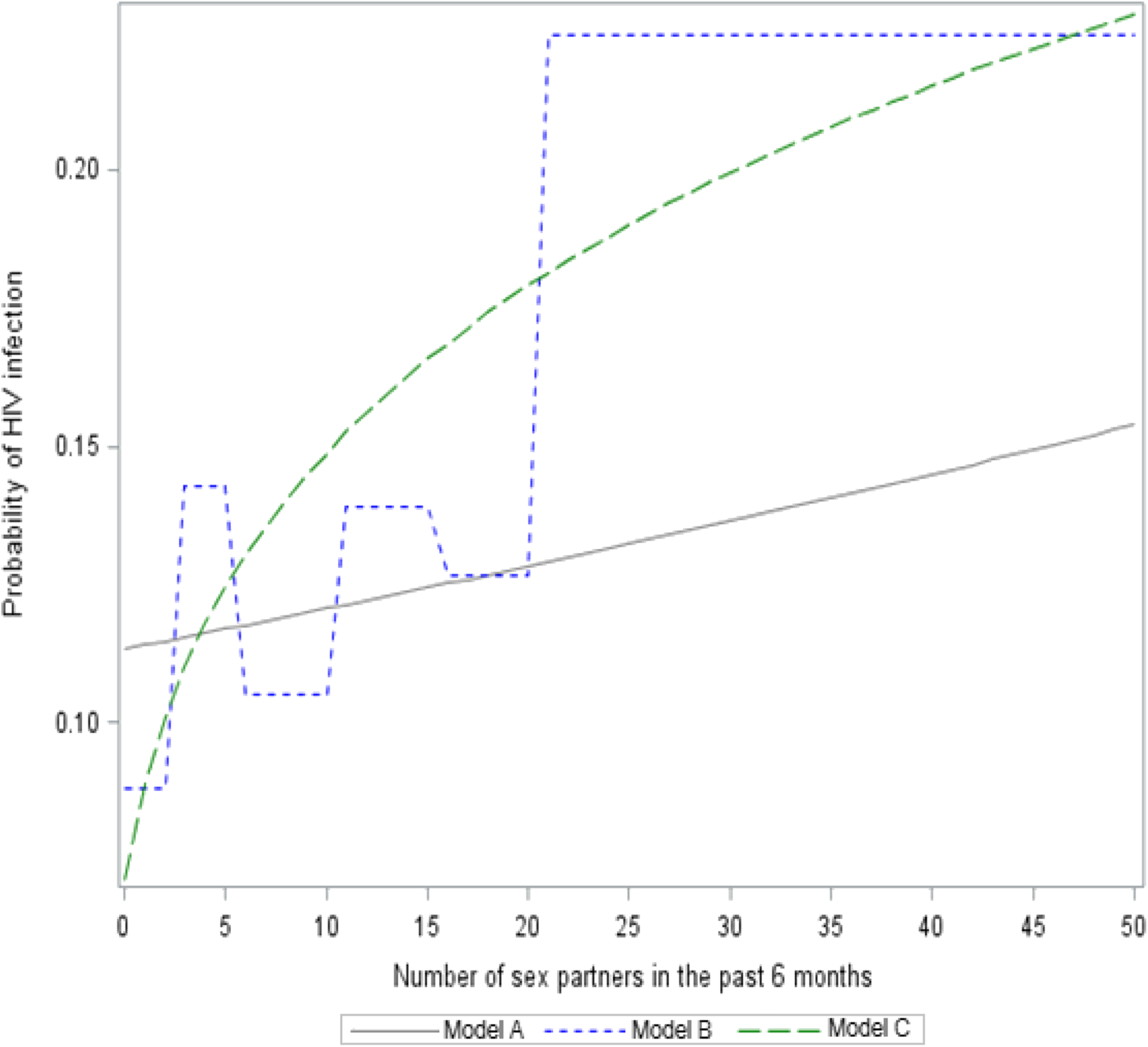

Supplement: Supplementary file 7 — Authors’ original file for figure 5 [file 12879_2014_3848_MOESM7_ESM.tif]
