# Supplementary material for: Risk of using logistic regression to illustrate exposure-response relationship of infectious diseases
Source: BMC Infect Dis. 2014 Oct 4;14:540. doi: 10.1186/1471-2334-14-540 (PMC4287313; doi:10.1186/1471-2334-14-540)
Supplement: Supplementary file 8 — Authors’ original file for figure 6 [file 12879_2014_3848_MOESM8_ESM.pdf]

Table 1: Means and standard deviations of 3000 sample statistics (HIV prevalence=10%)

Models		Parameters	Mean	Standard Deviation	Coefficient of Variation
**A (Ordinary)**		Intercept (α)	-5.24	0.10	-2%
		Coefficient (β)	0.23	0.01	4%
**B (Categorical)**		Intercept (α)	-0.66	0.41	-62%
	**0-2 partners**	Coefficient (β)	ref	ref	
	**3-5 partners**	Coefficient (β)	-0.14	0.29	-207%
	**6-10 partners**	Coefficient (β)	-0.14	0.28	-200%
	**11-15 partners**	Coefficient (β)	2.06	0.52	25%
	**16-20 partners**	Coefficient (β)	4.06	0.52	13%
	**21-25 partners**	Coefficient (β)	4.67	0.53	11%
	**26-30 partners**	Coefficient (β)	5.26	0.53	10%
	**31-35 partners**	Coefficient (β)	5.97	0.55	9%
	**36-40 partners**	Coefficient (β)	6.91	0.73	11%
	**>=41 partners**	Coefficient (β)	10.02	3.78	38%
**C (Log transformation)**		Intercept (α)	-10.95	0.46	-4%
		Coefficient (β)	3.46	0.16	5%

*Direct comparisons of the model coefficient were not possible due to the difference in units and transformations. So, coefficient of variation (CV) was used to demonstrate the dispersion of coefficient.
